# Modifiable risk factors for homebound progression among those with and without dementia in a longitudinal survey of community-dwelling older adults

**DOI:** 10.1186/s12877-021-02506-1

**Published:** 2021-10-18

**Authors:** Mia Yang, Nicholas Pajewski, Mark Espeland, Douglas Easterling, Jeff D. Williamson

**Affiliations:** 1 Department of Internal Medicine, Section on Gerontology and Geriatric Medicine, Wake Forest School of Medicine, 1Medical Center Blvd, Winston-Salem, NC 27157 USA; 2 Department of Biostatistics and Data Science, School of Public Health Sciences, Wake Forest School of Medicine, Winston-Salem, NC USA; 3 Department of Social Sciences and Health Policy, School of Public Health Sciences, Wake Forest School of Medicine, Winston-Salem, NC USA

**Keywords:** Risk factors, Homebound progression, Dementia, Mobility, Depression, Pain

## Abstract

**Background:**

Being homebound is independently associated with increased mortality but the homebound population is heterogeneous. In order to improve precision medicine, we analyzed potentially modifiable factors that contribute to homebound progression (from independent to needing assistance, to homebound), stratified by dementia status.

**Methods:**

Using National Aging and Trends Survey (NHATS), a nationally-representative, longitudinal annual survey from 2011 to 2017 (*n* = 11,528), we categorized homebound progression if one transitioned from independent or needing assistance to homebound, including competing risks of institutionalization or death between 2011 and last year of data available for each unique respondent. Using proportional hazards regression, we calculated hazard ratios of potentially modifiable risk factors on homebound progression.

**Results:**

Depressive symptoms, mobility impairment, and pain increased risk of homebound progression regardless of dementia status. Social isolation increased risk of homebound progression only among those without dementia at baseline.

**Conclusion:**

Future clinical care and research should focus on the treatment of depressive symptoms, mobility, and pain to potentially delay progression to homebound status.

## Background

There are estimated 2 million Americans who are homebound, based on the National Health and Aging Trends Study (NHATS), a cross-sectional, nationally representative sample of community-dwelling, non-institutionalized Medicare beneficiaries ages 65 or older [1]. Cognitive and/or physical functional limitations contribute to the homebound continuum, ranging from independence, needing assistance to leave the home, to rarely/never leaving the home (homebound) [1]. Medicare’s definition of homebound, associated with eligibility to receive home health services, means one requires physical or personal assistance to leave the home and that it requires a “taxing effort” [2], which aligns with both assisted and homebound categories within NHATS [1]. Homebound status in older adults indicates vulnerability to worse healthcare outcomes: it is independently associated with more than twice the risk of death, in addition to more comorbidities, more functional impairment, and dementia [1–3].

The prevalence of Alzheimer’s disease and related dementias (ADRD) is particularly high in older adults who are homebound. ADRD prevalence is strongly correlated with degree of homebound status: 80% of those who were homebound had dementia, versus 57% of those who needed assistance and 14.8% of those who were independent [1].

Older age, being female or Hispanic, social isolation, smoking, having dementia, history of falls, use of walking assistive devices, and depression/anxiety are all associated with increased risk of becoming homebound [4], [5], [6]. Longitudinal studies show conflicting data as to whether barriers at entry of the home were associated with becoming homebound [7], [8]. Other factors that are highly prevalent in the homebound population such as sensory impairment [9], pain [10], [11], and sleep [10] have not been examined as potential risk factors for homebound progression. Current epidemiologic studies of homebound people do not distinguish those who are homebound due to physical vs cognitive impairment [4], [12], [13], [14]. Therefore, it is essential to differentiate between individuals with dementia and those with normal cognition who are homebound, to achieve a more personalized approach to their clinical care. The purpose of this study was to determine which potentially modifiable factors contribute to homebound progression (from independent living to needing assistance to homebound), stratified by dementia status.

## Methods

### Study design

The National Health and Aging Trends Study sample uses US Medicare enrollment file as the sampling frame for selecting people age 65 and older enrolled in a given year (9/30/2010 for the original 2011 cohort and 9/30/2014 for the replenishment cohort in 2015). Due to cost of in-person interviews, only contiguous United States territories were sampled, excluding Alaska, Hawaii, and Puerto Rico [15]. NHATS was performed in accordance with the Declaration of Helsinki and approved by Johns Hopkins Institutional Review Board. Interviews were completed annually and resulted in 11,528 unique respondents from both cohorts. Two-hour in-person interviews were conducted to collect self- or proxy-reported physical activity, functional status, chronic health conditions, and economic status. Physical and cognitive test batteries were conducted. Proxy answers consisted of 5.4% of all respondents at their baseline. Annual attrition from NHATS varied between 12.7% (between 2015 to 2016) to 18% (2012–2013 and 2013–2014).

### Study population

NHATS oversampled of those over 90 years old and non-Hispanic blacks. Among 8245 participants in 2011, over 40% were ages 80 or older and about 20% self-identified as Black or African American [16]. Persons ages 65 and older not enrolled in Medicare or those who are ineligible for Medicare (such as immigrated to the US after age 65) are not sampled by NHATS [17]. In this study, we excluded nursing home respondents of NHATS in 2011 and in 2015 (baseline years for the original and replenishment cohorts) because of our focus on homebound progression and institutionalization as an important competing outcome.

### Dementia categorization

NHATS used three categories for cognitive status -- probable dementia, possible dementia (Mild Cognitive Impairment or MCI), or cognitively normal [18] based on 3 factors: prior diagnosis by a physician through sampled person or proxy’s self-reports, proxy scores on the AD8 Dementia Screening Interview (which includes temporal orientation, memory, judgment, and function tests), and additional in-person tests of memory, orientation, and executive function. Using the Aging, Demographics, and Memory Study (ADAMS) as a reference, the NHATS definition of dementia (probable and possible dementia combined) has a sensitivity of 85.7% for dementia. Specificity is higher for the narrow definition (probable vs no dementia) (87.2%) [18]. We used the narrow definition of dementia (probable dementia vs no dementia) in this study, as we wanted those with dementia to be clearly differentiated from those without dementia in terms of their potentially modifiable risk factors. We dichotomized dementia based on the narrow definition and compared risk factors within those who had dementia or not. We did not track progression of cognitive impairment to dementia over time in this analysis. We classified dementia status based on NHATS definitions of probable, possible, and no dementia for each respondent at baseline (either 2011 or 2015 replenishment cohort) [18].

### Homebound definition

NHATS has no pre-defined measure of homebound status. We used Ornstein’s definition of homebound status by creating frequency of respondents leaving home, whether respondent had difficulty leaving home, and whether help was required to leave home within the past month [1]. Our categorizations of homebound status into 3 categories: independent, assist, and homebound, correspond to Ornstein’s categorization of “not homebound”, “semi-homebound”, and “homebound [1].” Participants were not considered homebound if they could independently leave their home without cognitive or physical difficulty, such as wishing to stay home out of personal preference [1]. In order to distinguish those who are homebound due to physical vs cognitive impairment, we stratified this study based on dementia status. Those who are functionally limited due to cognition may have a unique set of risk factors compared to those who are only physically limited. We coded for homebound status within each round of NHATS and stacked round 1 through 7 in sequence.

### Outcome variables

A. **Primary outcome:** progression to homebound.

We identified progression to homebound as an ordinal survival analysis with repeated measures of NHATS respondents followed annually over the 7 waves of NHATS from 2011 to 2017. Since respondents could change from assisted to independent or homebound in consecutive years or improve in their function over time, we compared the homebound classification for the last year available for each respondent vs their baseline year’s homebound classification. Median follow-up years was 4, average 3.94 years, with minimum 1 year, maximum 7 years. Because being homebound is intrinsically linked to one’s degree of physical or cognitive impairment, institutionalization and death are important competing risks to those who continue to decline in function at home. If homebound individuals are not able to leave home due to physical and or cognitive impairment, they would also find institutionalization as an important patient-oriented outcome as patients prefer to remain in their own homes [19] the majority of the time.

Thus, risks of institutionalization or death were included as possible outcomes in the progression definition. Most progression events did not include institutionalization or death (2485/3330 or 75%).

B. Demographic data at baseline:

All covariates including age, gender, ethnicity/race, education level, and marital status, were based on the baseline assessment (either year 2011 or 2015 in the original or replenishment cohort, respectively).

C. **Potential risk factors (**Table 3**).**

We chose risk factors that were potentially modifiable from the perspective of a clinician. All risk factors were assessed at baseline (either 2011 or 2015). The replenishment cohort in 2015 was collected using the same methodology, allowing for analysis of trends over time. We classified individuals as having depressive symptoms if they responded that they lacked interest in usual activities or felt down or hopeless more than half of the days in the past month. We defined sleep problems needing over 30 min to fall asleep or having trouble falling back to sleep some nights, most nights, or every night, which has been associated with incidence dementia risk [20]. We defined social isolation in this study as having 2 or fewer persons in each respondent’s social network [21] since we adjusted for covariate of cohabitating with at least one person as marital status. Multimorbidity was defined as having at least 3 chronic conditions. Chronic conditions specifically asked by NHATS are: heart attack/myocardial infarction, heart disease, hypertension, arthritis, osteoporosis, diabetes, lung disease, stroke, dementia/Alzheimer’s disease, and cancer.

### Data sources and data processing

We used publicly available data from nhats.org and compiled each unique respondent’s sequential answers on consecutive years of NHATS’ surveys into a master file.

### Data analysis

We used Cox proportional hazard regression (SAS v. 9.4, Cary, NC) to analyze the risk of potentially modifiable variables on homebound progression. Models included adjustment for age, gender, race, marital status, and educational status. Potentially changeable predictor variables include environmental factors such as barrier at entry of home, home modifications, fear of falls or falls, depression, pain, poor sleep, multimorbidity, social isolation, and sensory (e.g. hearing or vision) impairments. When there were multiple questions for certain variables, such as falls or hearing impairment, we chose the questions with the least (i.e. < 10%) of missing data. We did this rather than attempting multiple imputation or developing consolidated constructs from multiple questions for simplicity and clarity. Among the modifiable variables, the variable environmental/home modification, which 35.9% respondents’ answers were missing, and pain limiting function (72.5% missing), and thus were excluded in the analysis.

## Results

Overall about 30% of cohort are from age 65–69, 23% from age 70–74, 18% from age 75–79, 14% from age 80–84, and 9% from age 85–89, and 5% from 90+. Women are slightly more represented at 57%. About 81% are non-Hispanic White, about 8.2% are non-Hispanic Blacks, about 4% are Hispanic, and about 6.5% are non-Hispanic Other. The cohort in NHATS are highly correlated with Medicare and census demographic of older adults in the US.

There were significant differences among those with dementia vs without dementia in age, gender, race/ethnicity, education, and marital status (Tables 1 & 2). Those with dementia were more likely to be older, female, non-Hispanic Black, Hispanic, or other ethnicities (Table 2). They were also more likely to have lower educational levels or live alone/separated (Table 2).

**Table 1:** Demographic factors related to homebound status among those cognitively normal at baseline, NHATS 2011 & 2015 (*n* = 11,528)

	Cognitively Normal Mean (SD) or Frequency (Percent)		
	Indep *N* = 8167	Assist *N* = 1496	HB *N* = 424
Age	75.6 (7.1)	78.9 (8.2)	81.0 (7.3)
Gender			
Female	3574 (46.0)	463 (31.0)	90 (21.2)
Male	4413 (54.0)	1033 (69.0)	334 (79.8)
Race/Ethnicity			
Non-Hispanic White	5803 (71.0)	947 (63.3)	239 (56.4)
Non-Hispanic Black	1594 (19.5)	368 (24.6)	110 (25.9)
Hispanic	399 (4.9)	108 (7.2)	60 (14.2)
Other/Missing	239 (3.4)	73 (4.9)	15 (3.5)
Education			
≥ College graduate	2069 (25.8)	227 (15.5)	41 (9.8)
High school graduate	4422 (55.1)	782 (53.4)	200 (47.8)
< High school graduate	1540 (19.2)	455 (31.1)	177 (42.3)
Marital status			
Married/Cohabitating	4598 (56.4)	573 (38.4)	113 (26.7)
Separated/Living Alone	3562 (43.6)	921 (61.6)	310 (73.3)

*Abbreviations: HB* Homebound, *Indep* Independent, *SD* Standard deviation

**Table 2:** Demographic factors related to homebound status among those with dementia at baseline, NHATS 2011 & 2015 (*n* = 11,528)

	Dementia Mean (SD) or Frequency (Percent)			Interaction w/ HB status
	Indep *N* = 518	Assist *N* = 525	HB *N* = 398	
Age	81.4 (7.3)	83.8 (7.9)	86.0 (7.6)	0.17
Gender				
Female	268 (51.7)	180 (35.8)	109 (27.4)	0.13
Male	250 (48.3)	337 (64.2)	289 (72.6)	
Race/Ethnicity				
Non-Hispanic White	284 (54.6)	280 (53.3)	197 (49.5)	
Non-Hispanic Black	144 (27.8)	154 (29.3)	125 (31.4)	< 0.001
Hispanic	52 (10.0)	49 (9.3)	46 (11.6)	
Other/Missing	38 (7.3)	42 (8.0)	30 (7.5)	
Education				
≥ College graduate	49 (9.8)	56 (11.20	39 (10.6)	
High school graduate	209 (42.0)	198 (39.6)	148 (40.2)	< 0.001
< High school graduate	240 (48.2)	148 (49.2)	181 (49.2)	
Marital status				
Married/Cohabitating	232 (44.9)	162 (30.9)	112 (28.4)	< 0.001
Separated/Living Alone	285 (55.1)	363 (69.1)	282 (71.6)	

NHATS respondents with dementia had higher prevalence of being homebound compared to those without dementia, 27.8% vs 8.2% (Table 3). Persons with dementia were also more likely to remain homebound (78.7% of those with dementia vs 56.4% of those without dementia). Persons with probable dementia were more likely to have progression events (55%) than those with possible dementia (39.2%) or those who were cognitively normal (22.7%, data not shown).

**Table 3:** Initial and final homebound status stratified by dementia status, NHATS 2011–2017 (*n* = 11,528)

	No Dementia Frequency (Percent)			Dementia Frequency (Percent)		
	Initially Independent	Initially Assist	Initially Homebound	Initially Independent	Initially Assist	Initially Homebound
Final Status Independent	5663 (68.1)	392 (22.2)	52 (7.8)	152 (41.9)	22 (8.6)	5 (3.2)
Final Status Assist	1029 (12.4)	678 (38.4)	129 (19.3)	110 (30.3)	135 (52.5)	28 (18.1)
Final Status Homebound	682 (8.2)	463 (26.3)	376 (56.4)	101 (27.8)	100 (38.9)	122 (78.7)
Total	8322	1764	667	363	257	155

Among those homebound due to dementia, depressive symptoms, using a cane/walker, having falls or worry about falls within the past month, and being bothered by pain were associated with increased risk of becoming homebound. Among those homebound and were cognitively normal, or due to physical impairments only, social isolation was significantly associated with increased risk of progression of homebound status (HR 1.15 (1.047, 1.256)). Those who had stairs to enter the home had slightly reduced risk of progression only among those who were cognitively normal (HR 0.91 (0.836, 0.998)).

Most of the risk factors influenced homebound progression regardless of dementia status: depressive symptoms, mobility impairments, falls, and being bothered by pain after adjusting for age, gender, race/ethnicity, marital status, and educational status (Table 4). Depressive symptoms increased the risk of homebound progression regardless of dementia status (interaction *p*-value 0.11). Mobility impairment (e.g., using a cane or walker) significantly increased risk of homebound progression regardless of dementia status (HR 1.97 for no dementia, HR 1.39 for dementia). However, use of assistive devices was more common among those with dementia (interaction *p*-value < 0.001). Both past history of falls or worry about falling were associated with increased risk of homebound progression, regardless of dementia status (HR 1.25–1.53). Being bothered by pain was associated with higher risk of homebound progression, regardless of dementia status (HR ≈ 1.2). Neither wearing glasses or hearing aids posed a significant subsequent risk for progression regardless of dementia status.

**Table 4:** Factors related to homebound progression based on proportional hazards regression among individuals living independently at baseline, with covariate adjustment for demographic factors, NHATS 2011–2017 (*n* = 11,528). *95% confidence interval excludes 1

Modifiable Factors	No Dementia HR [95% CI]	Dementia HR [95%]	Interaction ***p***-value
Wears glasses	1.080 [0.994,1.173]	1.052 [0.91,1.217]	0.85
Depressive symptoms	1.398 [1.266,1.544]*	1.184 [1.020,1.375]*	0.11
Wears hearing aid	0.938 [0.841,1.047]	0.910 [0.743,1.116]	0.31
Social isolation	**1.147 [1.047, 1.256]**	0.976 [0.764, 1.247]	0.23
Stairs to enter home	0.913 [0.836,0.998]*	1.011 [0.867,1.179]	0.06
Using cane/walker	1.968 [1.790,2.163]*	1.386 [1.172,1.639]*	**< 0.001**
Falls within past month	1.382 [1.229, 1.555]*	1.334 [1.126, 1.579]*	0.99
Worry about falls	1.525 [1.399, 1.662]*	1.254 [1.080, 1.454]*	**0.007**
Bothered by pain	1.198 [1.104, 1.300]*	1.218 [1.051, 1.411]*	0.99
Trouble sleeping	1.070 [0.986, 1.162]	0.977 [0.843, 1.133]	0.34

*Abbreviations: HR* Hazards Ratio, *CI* Confidence interval

## Discussion

### Principal findings

Among those without dementia, social isolation was found to be uniquely associated with increased risk of homebound progression. Social isolation is the objective deficit in connections to family, friends, or the community, which is distinct from loneliness, a subjective assessment that social relationships are lacking [22]. Social isolation is associated with increase in all-cause mortality, re-hospitalizations, nutritional risk, falls, dementia, and negative health behaviors such as heavy drinking, smoking, and being sedentary [23]. Our study is the first to find that having fewer than 3 people to talk about important things in life is associated with increased risk of becoming homebound, among those who are cognitively normal. This association of increased risk is relevant even after adjusting for living with someone. Although this study does not identify the exact mechanism of how social isolation increases the risk of becoming homebound, clinicians should focus on identifying social isolation and encouraging greater social engagement among those cognitively normal.

Regardless of dementia status, depressive symptoms, pain, and mobility impairments including falls increased the likelihood of homebound progression. Depression is present in 40–60% of those who are homebound [6], [1] and co-exists with dementia in about 20% of persons living with dementia [24]. Screening for and treating depression is important as it is associated with increasing risk of homebound progression. Signs of mobility limitation (e.g. using a cane or walker and recent falls) were strongly associated with an increased risk of homebound progression regardless of presence of dementia. Use of assistive devices among those without dementia was more highly associated with risk of homebound progression compared those with dementia, possibly because those with dementia become homebound primarily through cognitive rather than physical disability.

### Comparison to related studies

Sensory modifications such as glasses and hearing aids had no impact on homebound progression regardless of dementia subgroup, despite having a significant interaction with homebound status. This is in contrast to previous reports of self-reported vision and hearing impairment associated with shorter life expectancy and shorter duration of life without health problems [25]. Homebound progression may be multifactorial; if so, examining sensory impairments alone may reveal no significant increased risk. Future studies can examine the risk of multiple factors as a cluster at predicting homebound progression.

### Implication of the results

Among those without cognitive impairment, social isolation (having fewer than 3 people to discuss important things) was associated with increased risk of homebound progression. We could deploy this simple question in routine clinical practice to assess for social isolation. Social isolation is also associated with increased risk of dementia; conversely social engagement was protective of developing dementia [26]. Clinicians should encourage greater social connection to potentially prevent homebound progression. Other disciplines such as social workers and care managers could augment clinicians’ encouragement with referrals and connections with local senior centers or community organizations. Regardless of cognitive status, clinicians could potentially focus on screening and treating for depressive symptoms, mobility impairment, fall prevention, and pain control as common risk factors for homebound progression.

### Strengths and limitations of the study

Strengths of this study include using a nationally representative sample of community-dwelling older adults tracked longitudinally in terms of homebound progression. We also examined factors ranging from individual to environmental. The prospective data within NHATS allowed us to temporally examine baseline risk factors that precede the progression event. The dataset is generalizable and scaled to represent the entire older adults within the US. The identification of modifiable risk factors, depressive symptoms, mobility, and pain, allow clinicians to inquire and treat these potentially reversible conditions to possibly prevent future functional decline. Policy makers could potentially use this information to advocate for long-term home-based support services, such as home health aides paid by Medicaid, to help older adults remain at home longer with slower progression to homebound status, institutionalization, and death. In addition, our study findings may be tested in interventions that specifically focus on these risk factors to see whether systematic identification and treatment may delay progression to becoming homebound.

This study has its limitations. Only 5.4% of those NHATS interviews were from proxy, yet 12.5% of NHATS respondents were classified as having dementia after objective cognitive testing. Among those homebound and had dementia, only 30% of responses were from proxies. It is possible that proxy answers for persons living with dementia may be inaccurate. For example, how often has the respondent had little interest or pleasure in doing things may be difficult for a proxy to distinguish from apathy of dementia. Another limitation is that only baseline risk factors were captured in the analysis, but many factors identified can vary over time. Further, many questions were focused on immobility over the past month, leading to reversals of homebound status over time. In addition, depending on when the NHATS interview was completed, responses may not reflect seasonal changes that may reduce ability to leave the home without assistance.

## Conclusion

In conclusion, there are fewer than expected differences in risk factors for homebound progression between those with and without dementia. Depressive symptoms, mobility problems, and pain are key risk factors to target in future intervention trials to evaluate whether treatment of these modifiable risk factors translate to preventing progression to homebound status.

## Data Availability

The datasets generated and analyzed during current study are publically available at www.nhats.org

## Abbreviations

NHATS: National Health and Aging Trends Study
ADRD: Alzheimer’s disease and related dementias
US: United States
MCI: Mild Cognitive Impairment
ADAMS: Aging, Demographics, and Memory Study
HB: Homebound
Indep: Independent
SD: Standard deviation
HR: Hazards Ratio
CI: Confidence interval
